# Supplementary material for: Vitamin D Modified DSS-Induced Colitis in Mice via STING Signaling Pathway
Source: Biology (Basel). 2025 Jun 18;14(6):715. doi: 10.3390/biology14060715 (PMC12190092; doi:10.3390/biology14060715)
Supplement: Supplementary file 1 [file biology-14-00715-s001.zip › Supplementary Materials S1: 16s-report of gut microbiota/Groups/betadiv/pcoa_weighted_unifrac_analysis/Pcoa_unifrac12_boxplot_ellipse.pdf]

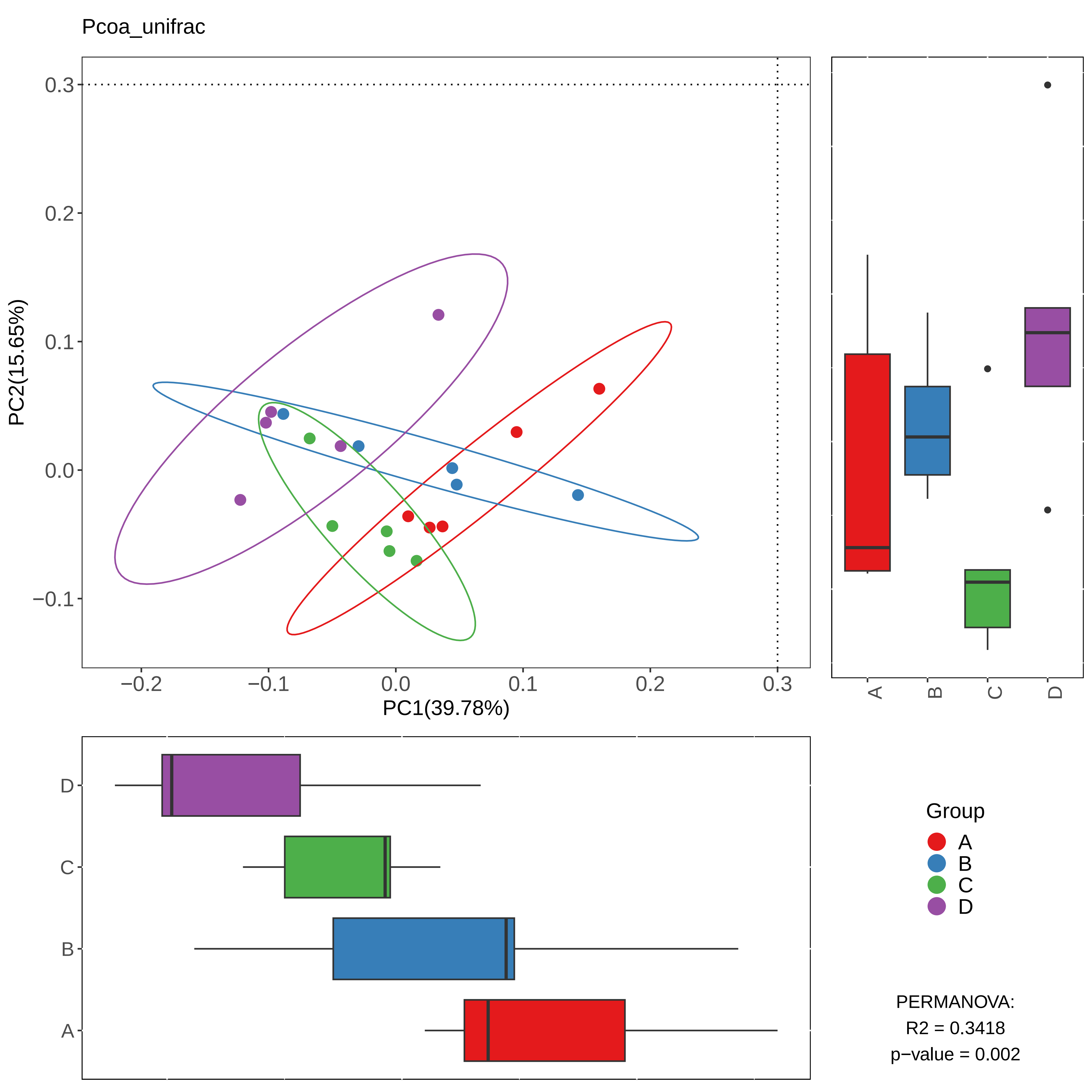

Pcoa_unifrac
0.3
0.2
0.1
0.0
−0.1
PC2(15.65%)
−0.2
−0.1
0.0
0.1
0.2
0.3
PC1(39.78%)
A
B
C
D
D
C
B
A
Group
A
B
C
D
PERMANOVA:
R2 = 0.3418
p−value = 0.002
